# Janus-micromotor-based on–off luminescence sensor for active TNT detection

**DOI:** 10.3762/bjnano.10.131

**Published:** 2019-06-28

**Authors:** Ye Yuan, Changyong Gao, Daolin Wang, Chang Zhou, Baohua Zhu, Qiang He

**Affiliations:** 1 Chemistry and Chemical Engineering College, Inner Mongolia University, College Road 235, Hohhot 010021, China; 2 Key Laboratory of Microsystems and Microstructures Manufacturing, Ministry of Education, Harbin Institute of Technology, Yi Kuang Jie 2, Harbin 150080, China

**Keywords:** autonomous motion, bubble propulsion, Janus micromotors, layer-by-layer assembly, TNT detection, upconverting nanoparticles (UCNPs), water pollution

## Abstract

An active TNT (2,4,6-trinitrotoluene) catalytic sensor based on Janus upconverting nanoparticle (UCNP)-functionalized micromotor capsules, displaying “on–off” luminescence with a low limit of detection has been developed. The Janus capsule motors were fabricated by layer-by-layer assembly of UCNP-functionalized polyelectrolyte microcapsules, followed by sputtering of a platinum layer onto one half of the capsule. By catalytic decomposition of hydrogen peroxide to oxygen bubbles, the Janus UCNP capsule motors are rapidly propelled with a speed of up to 110 μm s^−1^. Moreover, the Janus motors display efficient on–off luminescent detection of TNT. Owing to the unique motion of the Janus motor with bubble generation, the likelihood of collision with TNT molecules and the reaction rate between them are increased, resulting in a limit of detection as low as 2.4 ng mL^−1^ TNT within 1 minute. Such bubble-propelled Janus UCNP capsule motors have great potential for contaminated water analysis.

## Introduction

Water pollution has become a worldwide social problem. For example, the explosive TNT (2,4,6-trinitrotoluene), which is a highly toxic substance, has been widely used in military applications. The United States Environmental Protection Agency (USEPA) has classified TNT as hazardous waste, as it is possibly carcinogenic and mutagenic. Consequently, TNT-contaminated water has become one of the most serious pollution problems in war and military testing areas [1–7]. To date, various approaches including mass spectroscopy [8], ion transfer spectroscopy [9], surface plasmon resonance [10], molecularly imprinted polymers [6], and fluorescence polarization [11] have been proposed to detect TNT. However, most of these techniques have major limitations such as cumbersome pretreatment, complicated operation, long detection time and high cost. In recent years, owing to their simplicity, rare-earth-doped upconverting nanoparticles (UCNPs) have been developed for the detection of TNT [12]. Despite the advantages of UCNP-based TNT detection, it is still restricted by passive diffusion of TNT and UCNPs. Therefore, there is a significant need for the development of a fast and facile strategy to detect TNT that does not involve complicated sample pretreatment or expensive equipment.

Recently, synthetic micro/nanomotors have attracted tremendous attention because of their unique features and enormous potential applications in different fields [13–20]. Based on the concept of nanoarchitectonics [21–22], various kinds of micro/nanomotors have been fabricated, such as Janus capsule micromotors [23], tubular micromotors [24], helical nanomotors [25], nanowire motors [26], and nanorod motors [27]. Unlike inert particles that move by Brownian motion, micro/nanomotors can actively swim in solutions by converting energy from the environment (e.g., chemical fuel, light, acoustic or magnetic) into mechanical movement [28–37]. The active motion of micro/nanomotors has been proposed to improve reaction yields by overcoming the limitation of passive diffusion and enhancing the interaction between the reactants. However, self-propelled micromotors, to our knowledge, have never been explored for the detection of TNT.

Here, we report the first example of catalytic Janus capsule micromotors as luminescence quenching based sensors for active TNT detection. The Janus capsule micromotors were fabricated by depositing a thin platinum (Pt) film onto one hemisphere of the UCNP-functionalized hollow polyelectrolyte microcapsules. These as-prepared Janus micromotors can autonomously move by catalytic decomposition of hydrogen peroxide fuel into oxygen at a speed of up to 110 μm s^−1^ (22 body lengths per second). Meanwhile, the Janus microcapsules are able to actively adsorb and detect TNT based on the luminescence quenching of the UCNPs by TNT. The combination of efficient self-propulsion and TNT detection by these catalytic Janus micromotors demonstrates the potential for water pollutant analysis.

## Results and Discussion

**Figure 1:**
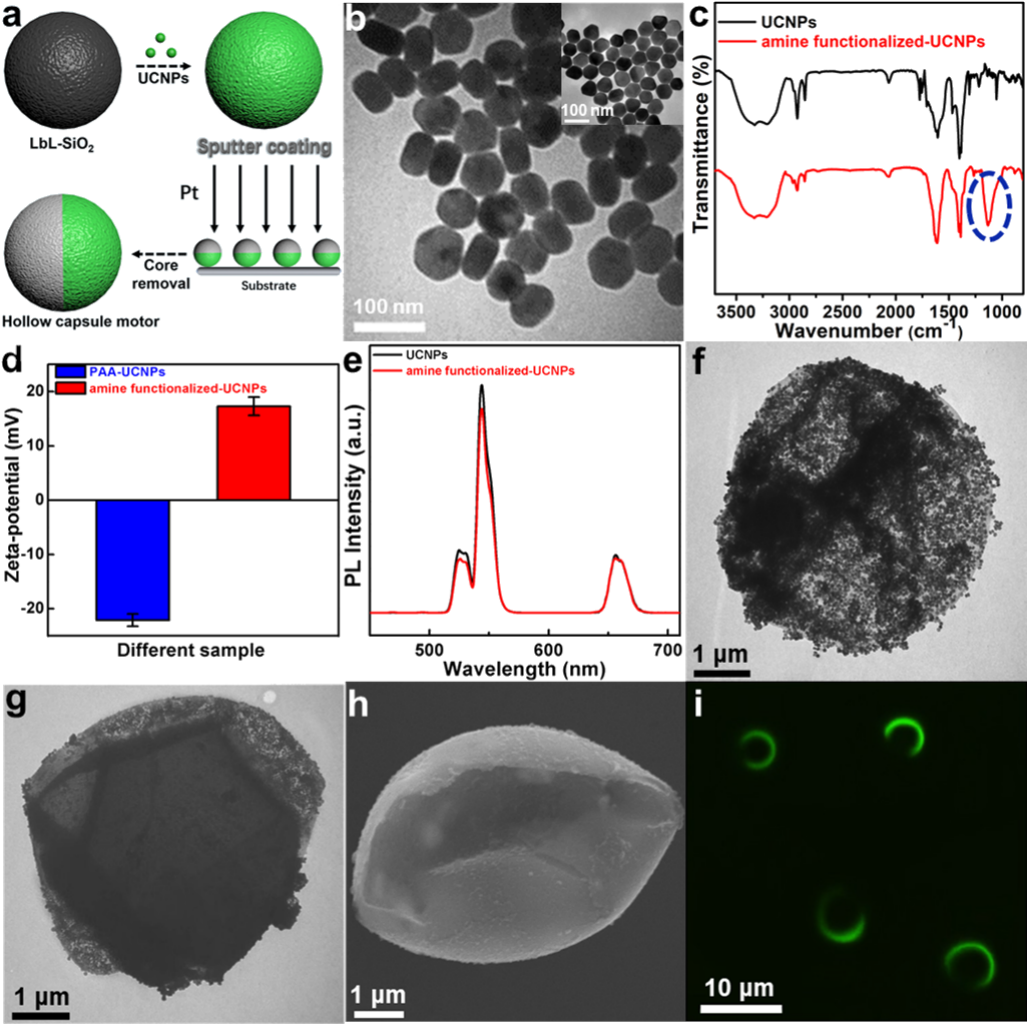
(a) Schematic representation of the fabrication process of Janus UCNP-modified polyelectrolyte capsule motors. (b) TEM images, (c) FTIR spectra, (d) zeta potential and (e) fluorescence emission spectrum of UNCPs with different surface functional groups. TEM images of UCNP-modified capsule (f) and Janus UCNP capsule motor (g). (h) SEM image of the Janus UCNP capsule motor. (i) Fluorescence microscope image of the Janus UCNP capsule motors.

The Janus UCNP-functionalized hollow polyelectrolyte capsule micromotors were prepared via a template-assisted method as schematically shown in Figure 1a. Briefly, eight bilayers of poly(allylamine hydrochloride) (PAH) and poly(styrene sulfonate) (PSS) were alternately deposited onto the surface of 5 μm silica particles by layer-by-layer (LbL) assembly [38–41]. The as-prepared particles were subsequently modified with amine-functionalized UCNPs through electrostatic interactions and then were dispersed on a glass slide to form a monolayer. After sputtering of a 20 nm thin film of Pt, the Janus UCNP-functionalized capsule motors were obtained by removing the silica cores with hydrofluoric acid.

To prepare the Janus UCNP capsule motors, NaYF_4_:Yb^3+^/Er^3+^ UCNPs were firstly synthesized following a previously published procedure with several modifications [42]. After treatment with poly(acrylic acid) (PAA), the surface of the UCNPs were functionalized with 3-aminopropyltriethoxysilane (APTES) to introduce the amine group. The nanostructure and morphology of the synthesized UCNPs were investigated by transmission electron microscopy (TEM). The TEM images in Figure 1b show that both the as-prepared UCNPs (inset image) and the APTES-modified UCNPs have good monodispersity and a hexagonal structure. The diameter of the APTES-UCNPs increased from 65 nm to 71 nm after modification with APTES. The chemical groups on the surface of the UCNPs were illustrated by Fourier-transform infrared (FTIR) spectroscopy. Figure 1c shows the FTIR spectra of the UCNPs and APTES-UCNPs. Compared with the FTIR spectrum of unmodified UCNPs, a notable transmission band peak at 1128 cm^−1^ (blue circle), attributable to the Si–O stretching vibration, can be seen in the FTIR spectra of APTES-UCNPs. These results indicate that the UCNPs were successfully modified with APTES. It has been demonstrated that the amine group is important to allow UCNPs to detect TNT. To verify the functionalization of the APTES-UCNPs with amine groups, the surface charge of the UCNPs before and after modification was measured. As shown in Figure 1d, the zeta potential changed from −22.08 to 17.3 mV, indicating the successful surface amine group functionalization. Moreover, the fluorescence emission spectrum shows that the surface functionalization did not affect the photoluminescence properties of the UCNPs (Figure 1e).

Furthermore, the structure and morphology of the Janus UCNP capsule motors were systematically characterized. The TEM images in Figure 1f show a microcapsule after functionalizing with UCNPs. It is evident that the APTES-UCNPs were uniformly dispersed on the surface of the microcapsule. After Pt sputtering and removal of the silica core, the Janus UCNP capsule motors were obtained. As shown in Figure 1g, the as-prepared APTES-UCNPs were partially covered with a Pt cap, displaying a Janus structure. The scanning electron microscopy (SEM) image in Figure 1h further confirms the asymmetric distribution of Pt on the one side of the Janus UCNP capsule motors. Due to the partial coverage of the Pt layer, the Janus UCNP capsule motors show a semicircle shape under excitation with a 980 nm laser (Figure 1i). These results confirm that the Janus UCNP capsule motors were successfully prepared.

**Figure 2:**
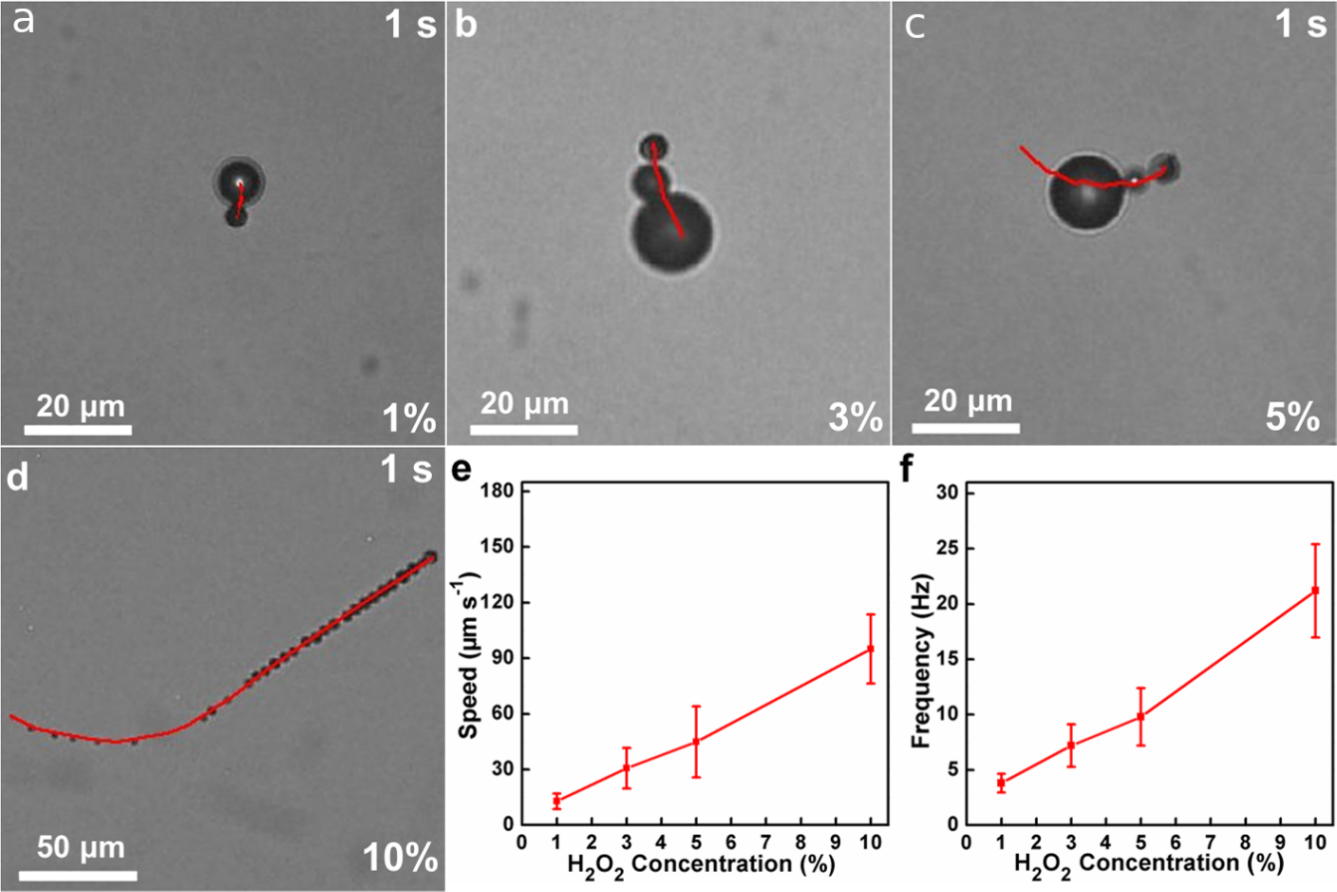
Images of the Janus UCNP capsule motors for different H_2_O_2_ concentrations: (a) 1%, (b) 3%, (c) 5% and (d) 10% after a period of 1 s. (e) Effect of the H_2_O_2_ concentration on the average speed of the Janus UCNP capsule motors. (f) Relationship of the oxygen bubble expulsion frequency of Janus UCNP capsule motors with different H_2_O_2_ concentration.

The autonomous motion of the Janus UCNP capsule motors was recorded by using microscopy. To better explore the movement behaviour of the Janus UCNP capsule motors, the corresponding motion trajectories, speed, and oxygen bubble frequency were studied systematically. The typical time-lapse images in Figure 2a–d (taken from the corresponding video in Supporting Information Files 1–4) show the trajectories of the motors in H_2_O_2_ solution with a concentration of 1%, 3%, 5% and 10% over a period of 1 s, respectively. It could be obviously seen that the Janus UCNP capsule motors swim in the H_2_O_2_ solution under the propulsion of oxygen bubbles that are generated by the catalytic decomposition of H_2_O_2_ fuel on the Pt side. The dependence of the H_2_O_2_ fuel concentration on the average speed of the motors was investigated. As shown in the Figure 2e, the average speed of the motors increased with increasing concentration of H_2_O_2_. It can be found that the average speed of the Janus UCNP capsule motors increases from ≈13 µm s^−1^ (≈2.6 body lengths s^−1^) at 1% H_2_O_2_ to ≈110 µm s^−1^ (≈22 body lengths s^−1^) at 10% H_2_O_2_. By analysing the released oxygen bubbles, we also obtained the relationship between the bubble expulsion frequency and the speed of the Janus UCNP capsule motors (Figure 2f). We found that the bubble expulsion frequency exhibits a similar trend with the concentration of H_2_O_2_, indicating a positive correlation between the oxygen bubble expulsion frequency and the speed of the motors. Taken together, these results demonstrate that the obtained Janus UCNP capsule motors have desirable motion capability and their movement speed can be controlled by adjusting the concentration of H_2_O_2_ and the expulsion frequency of the oxygen bubbles.

**Figure 3:**
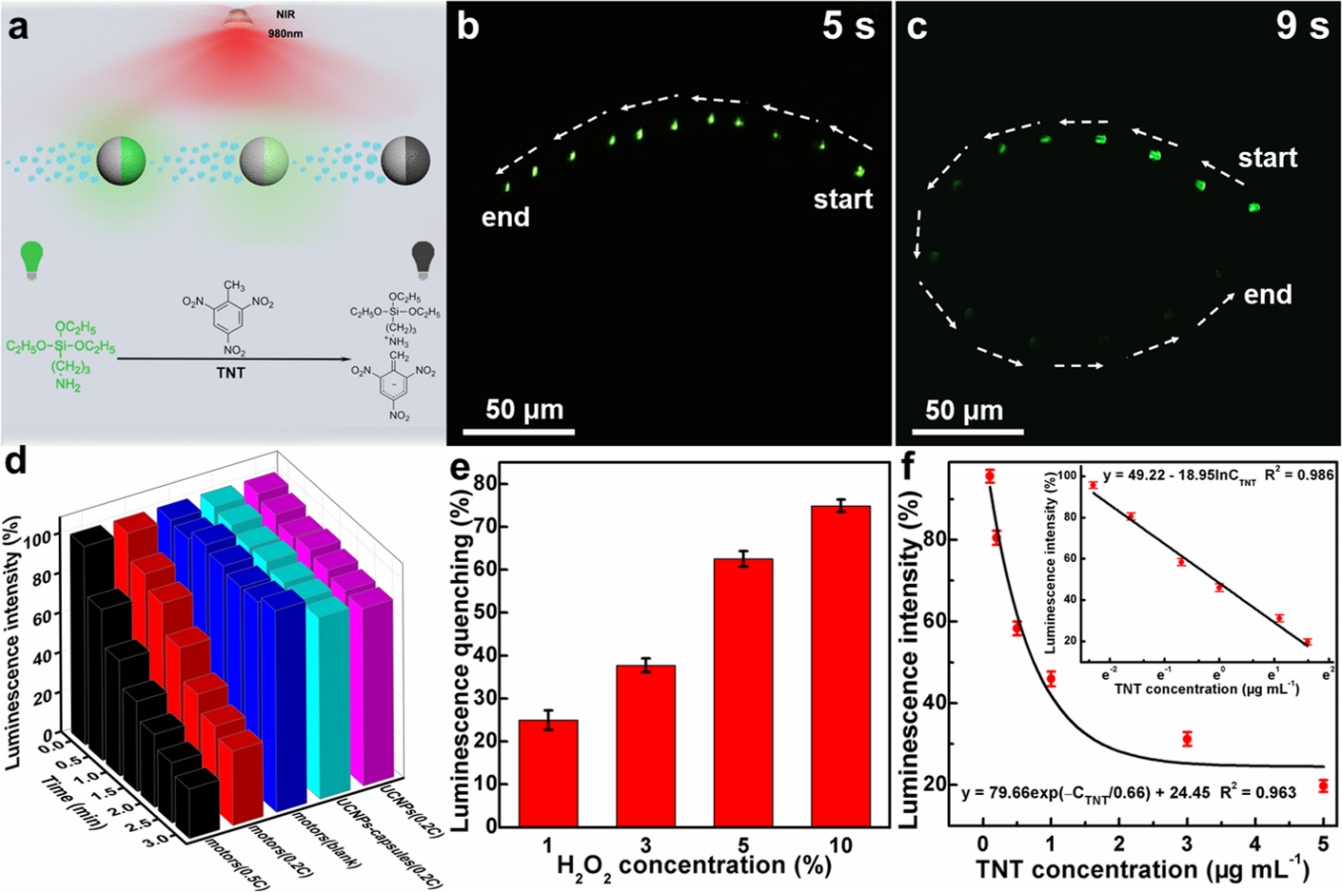
(a) Schematic illustration of the on–off luminescent detection of TNT by the Janus UCNP capsule motors. (b) Time lapse image of a Janus UCNP capsule motor in 5% H_2_O_2_ without TNT in 5 s under a 980 nm laser excitation. (c) Time lapse image of a Janus UCNP capsule motor in 5% H_2_O_2_ with 0.5 mg mL^−1^ of TNT in 9 s under 980 nm laser excitation. (d) Time-dependent luminescence quenching of different samples in TNT solution with different concentrations (*c* = 1 μg mL^−1^). (e) Plot of the luminescence quenching effect of Janus UCNP capsule motors in different H_2_O_2_ concentrations with a TNT concentration of 0.2 μg mL^−1^ for 3 min. (f) Luminescence intensity of a Janus UCNP capsule motor after autonomous movement for 1 min in a TNT solution in response to the TNT concentration and the resultant fitting equation. The inset demonstrates a linear relationship between the luminescence intensity of the Janus UCNP capsule motors and the natural logarithm of the TNT concentration.

Figure 3a schematically illustrates the Janus UCNP capsule motor based, real time luminescence on–off detection of TNT. The luminescence quenching mechanism involves the Janus UCNP capsule micromotor and TNT. Owing to the active motion of the motors, the amino groups of the PAA chains modified on the surface of the UCNPs could chemically recognize the TNT molecules efficiently and form a Meisenheimer complex which has a strong absorption within the emission spectrum of the UCNPs. Due to the fluorescence resonance energy transfer from the excited UCNPs to the complex, the luminescence intensity of the Janus UCNP capsule motor is reduced, simplifying the visual detection of TNT. To assess the TNT detection capacity of the motors, they were dispersed in 5% H_2_O_2_ with and without TNT at pH 12. It was found that the upconversion luminescence of the Janus UCNP capsule motor exhibits no obvious change in movement in 5% H_2_O_2_ without TNT (Figure 3b and Video Supporting Information File 1). The time lapse image in Figure 3c (captured from the video in Supporting Information File 2) demonstrates that the upconversion luminescence of the Janus UCNP capsule motor decreased gradually with via self-propelled movement in 5% H_2_O_2_ with 0.5 mg mL^−1^ of TNT, indicating that the green upconversion luminescence (543 nm) of the UCNPs was quenched by the presence of TNT.

The TNT sensing capability of these Janus UCNPs capsule motors was further evaluated by measuring the luminescence intensity. Figure 3d illustrates the time-dependent luminescence response of Janus UCNP capsule motors in TNT-contaminated H_2_O_2_. The motors were self-propelled for 3 min in 5% H_2_O_2_ containing 0.2 *c* (here, *c* = 1 μg mL^−1^) TNT and showed instant luminescence quenching, while both the UCNPs and static Janus UCNP capsule motors displayed no obvious luminescence quenching under the same conditions. This finding demonstrates that the TNT detection efficiency of active Janus UCNP capsule motors is higher than that of the passive UCNPs and UCNP capsules owing to the continuous fluid mixing and the enhanced real time reactions between TNT and the Janus UCNP capsule motors [43]. Furthermore, the luminescence quenching effect of the Janus UCNP capsule motor is more obvious in TNT solutions with higher concentration. We also found that the TNT detection efficiency of the Janus UCNP capsule motors increases with the increase of the H_2_O_2_ concentration (Figure 3e).

To determine the relationship between the TNT concentration and the luminescence quenching of the motors, they were dispersed in 5% H_2_O_2_ containing TNT with a concentration from 0 to 5 μg mL^−1^. After motion for 1 min, the luminescence quenching of the Janus UCNP capsule motor was tested using a fluorescence spectrometer. As shown in Figure 3f, the luminescence quenching of the motor firstly presents a linear relationship with the increase of the TNT concentration and then decays exponentially. The specific relationship can be fitted to the formula *y* = 79.66 exp(−*c*_TNT_/0.66) + 24.45. The inset image in Figure 3d shows that a linear relationship exists between the luminescence intensity of the Janus UCNP capsule motors and the natural logarithm of the TNT concentration. The corresponding limit of detection (LOD) is calculated to be 2.4 ng mL^−1^ according to the equation LOD = (3SD)/*k*, where the SD is the standard deviation of the luminescence intensity of the Janus UCNP capsule motors and the *k* is the slope of the calibration curve (*k* = 18.95). Compared with the LOD of passive UCNPs (8.4 ng mL^−1^), the TNT detection efficiency is enhanced by 3.5 times.

## Conclusion

We have developed a micromotor-based active sensor for the detection of TNT based on the on–off luminescence of a Janus UCNP capsule motor. The Janus UCNP capsule motors were fabricated by layer-by-layer assembly combined with vacuum deposition. These Janus motors with a catalytic Pt layer can be propelled by oxygen bubbles at a speed of up to 110 μm s^−1^ in 10% H_2_O_2_ fuel. Owing to their active motion capability, the as-prepared Janus motor could quickly absorb TNT molecules. More importantly, the Janus UCNP capsule motor can effectively detect TNT based on the on–off luminescence of amine-functionalized UCNPs. Benefiting from the enhanced autonomous motion, the LOD of the Janus UCNP capsule motors (2.4 ng mL^−1^) is 3.5 times better than that of static UCNPs within shorter analysis time. Such a micromotor could be used as a multifunctional platform integrating autonomous motion and TNT detection for efficient and rapid detection of environmental pollutants.

## Experimental

### Materials

Silica spheres with a diameter of 5 µm were obtained from Baseline Chrom Tech, Tianjin, China. Poly(styrene sulfonate) sodium salt (PSS, *M*_w_ = 70 000), poly(allylamine hydrochloride) (PAH, *M*_w_ = 70 000), poly(acrylic acid) (PAA, *M*_w_ = 1800), Y(NO_3_)_3_·6H_2_O, Er(NO_3_)_3_·6H_2_O, and Yb(NO_3_)_3_·6H_2_O were purchased from Sigma-Aldrich. 2,4,6-Trinitrotoluene (TNT) was purchased from Best-reagent, Chengdu, China. 3-Aminopropyltriethoxysilane (APTES), ethylene glycol dimethacrylate (EGDMA) 2,2’-azobisisobutyronitrile (AIBN), 1-octadecene (ODE, 90%) and oleic acid (OA, 90%) were obtained from Aladdin Chemistry Co. Ltd. NaOH, NH_4_F, ethanol, methanol, cyclohexane, trichloromethane, acetonitrile, NaCl, Na_2_HPO_4_, and NaH_2_PO_4_ were purchased from Tianjin Tianli Chemical Reagent Co. Ltd. Hydrofluoric acid (HF) and hydrogen peroxide (H_2_O_2_) were obtained from Beijing Chemical Works, China. All commercial materials were used without further purification.

### Preparation of Janus UCNP capsule motors

NaYF_4_:Yb/Er upconversion nanoparticles were firstly fabricated using a previously published method [42]. The obtained UCNPs (100 mg) were then mixed with PAA (300 mg) in 10 mL ethanol/chloroform (1:1) solution for 12 h at the room temperature. Then, the as-prepared PAA-UCNPs (80 mg) were added into 20 mL ethanol/acetonitrile (1:1) solution containing APTES (12.5 μL), EGDMA (25 μL), and AIBN (25 mg). After heating to 45 °C for 6 h, the amine-group-functionalized UCNPs were obtained.

To prepare Janus UCNP capsule motors, eight bilayers of PAH/PSS were deposited onto the surface of 5 μm silica particles through layer-by-layer assembly. Then, 1 mg of (PAH/PSS)_8_-coated particles were mixed with 10 mg of amine-group-functionalized UCNPs for 1 h under continuous shaking, and excess UCNPs were removed by centrifugation. The Janus structure was prepared by depositing a monolayer of UCNP-coated silica particles on glass substrates and sputtering 20 nm of Pt onto the surface of the UCNP-coated silica particles, followed by peeling off the particles from the substrate. The silica cores were then dissolved by treating the particles with 0.5 M HF. The Janus UCNP capsule motors were obtained after three centrifugation/water washing steps.

### Analysis of the movement of the Janus UCNP capsule motors

The movement studies were accomplished by dropping Janus UNCP capsule motors into hydrogen peroxide solutions of different concentration (1–10 vol %). The self-propelled motion of Janus UCNP capsule motors was recorded by using an Olympus BX53 fluorescence microscope. The trajectories of the motor movement were tracked by using the software of Image J and the motion velocity was analyzed using Origin 8 software.

### Characterization

SEM imaging was carried out by dropping sample solutions onto the surface of silicon wafer. After drying, samples were observed using a Hitachi S-5200 microscope. For TEM observation, samples were dropped onto the carbon film of the copper grids and observed using a Hitachi H-7650 microscope. UV−vis absorption spectra were recorded using a Hitachi U-4100 spectrophotometer. FTIR spectra were collected in the wavelength range from 4000 to 500 cm^−1^ by a Thermo Fisher 4700 Fourier transform infrared spectrophotometer with the KBr method. Upconversion luminescence was measured using a HORIBA Jobin Yvon FluoroMax-4 spectrophotometer with a 980 nm diode laser at a power of 1 W cm^−2^ (Shanghai Laser & Optics Century Co. Ltd).

## Supporting Information

File 1 The motion of a Janus UCNP capsule motor in 1% H_2_O_2_ solution.

File 2 The motion of a Janus UCNP capsule motor in 3% H_2_O_2_ solution.

File 3 The motion of a Janus UCNP capsule motor in 5% H_2_O_2_ solution.

File 4 The motion of a Janus UCNP capsule motor in 10% H_2_O_2_ solution.

File 5 The motion of a Janus UCNP capsule motor without luminescence quenching in 5% H_2_O_2_.

File 6 The on–off luminescence detection of TNT using a Janus UCNP capsule motor.
